# Efficacy of a Combined Acceptance and Commitment Intervention to Improve Psychological Flexibility and Associated Symptoms in Cancer Patients: Study Protocol for a Randomized Controlled Trial

**DOI:** 10.3389/fpsyg.2022.871929

**Published:** 2022-05-18

**Authors:** Francisco García-Torres, Ángel Gómez-Solís, Sebastián Rubio García, Rosario Castillo-Mayén, Verónica González Ruíz-Ruano, Eliana Moreno, Juan Antonio Moriana, Bárbara Luque-Salas, María José Jaén-Moreno, Fátima Cuadrado-Hidalgo, Mario Gálvez-Lara, Marcin Jablonski, Beatriz Rodríguez-Alonso, Enrique Aranda

**Affiliations:** ^1^Department of Psychology, University of Córdoba, Córdoba, Spain; ^2^Maimonides Biomedical Research Institute of Cordoba (IMIBIC), Córdoba, Spain; ^3^Reina Sofía University Hospital of Cordoba, Córdoba, Spain; ^4^Department of Specific Didactics, University of Córdoba, Córdoba, Spain; ^5^Department of Social Health Sciences, Radiology and Physical Medicine, University of Córdoba, Córdoba, Spain; ^6^Collegium Medicum Jan Kochanowski, University in Kielce, Kielce, Poland; ^7^Department of Medical Oncology, Reina Sofía University Hospital, Córdoba, Spain

**Keywords:** cancer, oncology, acceptation and commitment therapy, mhealth, psychological flexibility

## Abstract

Psychological flexibility is a key concept of acceptation and commitment therapy (ACT). This factor has been linked with psychological wellbeing and associated factors, such as quality of life, in cancer patients. These and other positive results of acceptation and commitment therapy in cancer patients found in previous research could be enhanced by using mhealth tools. A three-arm randomized superiority clinical trial, with a pre-post-follow-up repeated measures intergroup design with a 1:1:1 allocation ratio is proposed. A hundred and twenty cancer patients will be randomly assigned to one of the following interventions: (1) face-to-face ACT + mobile application (app), (2) face-to-face ACT, and (3) Waitlist control group. The primary expected outcome is to observe significant improvements in psychological flexibility acceptance and action questionnaire- II (AAQ-II) in the face-to-face ACT + app group, after comparing baseline and post-treatment scores, and the scores will remain stable in the two assessment points, 3 and 6 months after the intervention. Secondary expected outcomes are significant increasing scores in quality of life (EORTC QLQ C-30) and post-traumatic-growth (PTGI-SF), and significant decreasing scores in anxiety and depression (HADS), insomnia (ISI) and fatigue (BFI) at the same assessment points. Also, it is expected that the scores of this group will be higher than the scores of the face-to-face ACT group and the waitlist control group. This study aims to assess the efficacy of a combined intervention (face-to face ACT + app) for psychological flexibility and associated symptoms in cancer patients. The results of this protocol may help to consider the use of acceptation and commitment therapy and mhealth applications in cancer settings as a valid therapeutic choice.

**Clinical Trial Registration:**

[www.ClinicalTrials.gov], identifier [NCT05126823].

## Introduction

Cancer continues to be a relevant health problem worldwide. Despite advances in diagnostic, prevention and treatment techniques in recent years, cancer continues to appear as one of the leading causes of mortality worldwide, only behind cardiovascular disease (World Health Organization, 2021). Specifically, cancer caused 9.96 million deaths in 2020, with lung (18%) and colorectal cancer (9.4%) having the highest mortality rates. This pattern was also observed in Spain, where 113.054 people died of cancer, with lung and colorectal cancer having the highest mortality rate. Regarding the incidence, estimates predict that in the coming years a very significant increase in cases (+49.2%) will be observed worldwide, going from an estimated incidence of 18.094.716 in 2020 to 26.997.577 in 2040. In Spain, the data show a relatively stable trend with ascending values, since in 2021, for example, 276.239 cases were diagnosed and by 2022 it is estimated that 280.100 new cases will be diagnosed, but this data are probably influenced by the negative impact of the COVID-19 pandemic during this period. Despite this, the higher incidence rates correspond to colorectal (43.370), breast (34.750), and lung cancer (30.948) (Sociedad Española de Oncología Médica, 2022).

In cancer patients, it is common to observe certain impairments as a consequence of the disease and its treatments. The most frequent psychological symptoms are anxiety (10–18%) and depression (11–20%), which, in addition, are consistently related to a worse quality of life in this group of patients (Satin et al., 2009; Kuhnt et al., 2016; Pitman et al., 2018; De Mol et al., 2020; Erim et al., 2020; Park et al., 2020; Taarnhøj et al., 2020). Fatigue, understood as a subjective state of emotional and physical fatigue that interferes with the patient’s normal functioning, appears in up to 60% of cancer patients, while sleep disturbances, both in the form of insomnia, as in terms of difficulties when falling asleep, and maintaining sleep, is also observed in a high number of patients, in a range between 18 and 68% (Mock et al., 2000; Corbett et al., 2016; Zhou et al., 2017; Yun et al., 2021).

The acceptance and commitment therapy (ACT, Hayes et al., 2011) has shown to be an effective treatment for reducing these psychological symptoms and, thus, beneficial for improving psychological wellbeing in cancer patients. A central principle of ACT is to consider that, while suffering is unavoidable, it can be coped with by using psychological flexibility, defined as “contacting with the present moment as a conscious human being, fully and without needless defense-as it is and not as what it says it is-and persisting with or changing a behavior in the service of chosen values” (Hayes et al., 2011, p. 96–97). Specifically, the intervention with ACT focuses on three mechanisms that contribute, through psychological flexibility, to a healthy adjustment during the course of the disease in cancer patients: (1) being present in the here and now, (2) being opened to contact with emotions and (3) do what matters according to personal values (Fashler et al., 2018). In contrast to other psychological treatments, such as the cognitive behavioral therapy, ACT focuses on changing the patient’s relationship with their negative thoughts, without trying to change these thoughts. In fact, it is common for cancer patients that negative thoughts about the disease, the treatment or the prognosis might be realistic and coherent with their current state and not the product of distorted information processing. In this way, encouraging patients to change the relationship they have with their thoughts rather than trying to modify them can have a positive effect on patients’ adjustment to the disease (Hulbert-Williams et al., 2015; Fashler et al., 2018).

In a recent systematic review of randomized and controlled trials on the efficacy of the use of ACT in cancer patients, applied individually or in groups, the authors observed that this therapy has shown to be effective for reducing symptoms of anxiety, depression, sleep problems, and pain, among others (Bas and Dirik, 2019). A similar review concluded that applying ACT was beneficial for increasing the psychological flexibility of cancer patients, in addition to obtaining improvements in their quality of life and emotional state (González-Fernández and Fernández-Rodríguez, 2019). In this sense, psychological flexibility appears as a key element to explain the therapeutic change after the application of ACT in cancer patients. The results obtained by Aguirre-Camacho and Moreno-Jiménez (2017) suggest that psychological flexibility is the mediating variable of the positive effects of face-to-face interventions in ACT on anxiety, depression and quality of life in cancer patients. These results are supported by different studies in samples with various types of cancer (Feros et al., 2013; Arch and Mitchell, 2016), which highlights the need to consider the psychological flexibility as a therapeutic target in this group of patients, with the aim of improving their emotional adjustment and quality of life. However, despite these promising results, these authors have emphasized that more empirical support from controlled trials with adequate follow-up is needed to establish the efficacy of ACT in cancer patients.

Although the use of non-face-to-face ACT-based interventions might be useful for patients with difficulties in accessing health centers, there is still little evidence of the benefits of this therapeutic modality for cancer patients. The previous literature in this regard shows the positive result of the randomized clinical trial carried out by Hawkes et al. (2014), in which the authors used the telephone as a means to carry out an intervention based on symptom management, coping with stress and training in a healthy lifestyle, compared to a control group that received the usual treatment. The results showed that patients assigned to the ACT group had improvements in emotional wellbeing compared to the group that received no treatment. In a similar intervention, using the telephone as a means to carry out an ACT-based intervention, Mosher et al. (2018) observed that after finishing it, improvements were obtained in the level of depression and anxiety, in addition to lower levels of fatigue and sleep problems in patients with metastatic breast cancer. Therefore, there is evidence that supports the use of non-face-to-face ACT-based interventions in cancer patients, since this format improves results when the interaction with the therapist is allowed (Baumeister et al., 2014).

Relatedly, Arch et al. (2020) carried out a randomized pilot trial with 35 stage IV patients with cancer, applying a combined ACT intervention (4 face-to-face sessions + online sessions), observing that after the intervention the levels of anxiety and depression were significantly reduced. In this line, Feros et al. (2013), using a combined ACT intervention (face-to-face + Compact Disk), in a research design with 28 patients with cancer and without a control group, observed that after the intervention, the patients obtained significant improvements in anxiety, depression, quality of life and psychological flexibility. Despite these positive results, the aforementioned authors highlighted the need to carry out studies with greater control (randomized and controlled trials), a larger sample size and with adequate follow-up to be able to establish the efficacy of the ACT in this group of patients.

In recent years there has been an increase in the use of mhealth applications (apps) in the context of cancer. The massive use of smartphones and the internet favors that users of health systems have direct access to information on aspects related to cancer (prevention, detection, treatment options, etc.), which results in different benefits for patients cancer, as better self-efficacy and empowerment of patients (Charbonneau et al., 2020). In a recent review on the use of apps in cancer patients, the authors concluded that the use of these apps has beneficial effects on symptom control; however, there is little evidence of the positive effect of the use of apps on other outcomes in studies that include interventions (Osborn et al., 2020).

Considering the previously exposed, this study has the following objectives: First, to test the efficacy of a combined psychological intervention (ACT face-to-face + app), in comparison with an ACT face-to-face intervention and a waitlist group, to increase psychological flexibility and to significantly improve scores for anxiety, depression, quality of life, fatigue, insomnia, and post-traumatic growth from baseline to end of the intervention and in the two follow up periods (3 and 6 months after the intervention). And, secondly, to test the efficacy of the face-to-face ACT-based intervention group compared to the waitlist control group in the mentioned variables, to provide further empirical support for the use of ACT in cancer patients.

Consequently, this study has the following hypotheses:

1. The combined intervention (face-to-face ACT + app) will be more effective in increasing psychological flexibility, quality of life and post-traumatic growth and reducing symptoms of anxiety, depression, fatigue and insomnia than the face-to-face ACT intervention at posttreatment and at the two follow-up periods (3 and 6 months).
2. The face-to-face ACT + app group will achieve significant improvements in psychological flexibility, quality of life and post-traumatic growth and significant reductions in anxiety, depression, fatigue, and insomnia compared to the waitlist group after the intervention and at the two follow-up periods (3 and 6 months).
3. The face-to-face ACT intervention group will significantly increase psychological flexibility, quality of life and post-traumatic growth, and reduce symptoms of anxiety, depression, fatigue, and insomnia compared to the waitlist group after the intervention and at the two follow-up periods (3 and 6 months).

## Methods and Analysis

### Study Design and Settings

**FIGURE 1:**
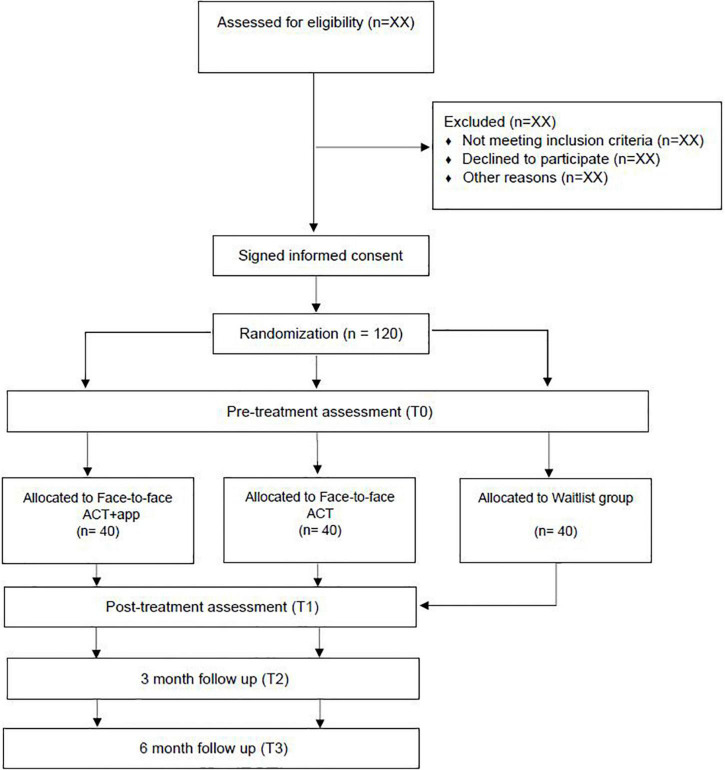
Study protocol flowchart.

This study is a three-arm randomized superiority clinical trial, with a pre-post-follow-up repeated measures intergroup design with a 1:1:1 allocation ratio. Participants will be randomly assigned to one of the following interventions: (1) face-to-face ACT + app, (2) face-to-face ACT and (3) Waitlist control group. For ethical reasons, the participants in the waitlist control condition will be offered the option to receive the treatment protocol. The evaluation will be carried out in four time points: initial evaluation before randomization (pre-treatment; T0), at the end of the intervention (post-treatment; T1), at 3 months after finishing the treatment (T2) and at 6 months after completing the intervention (T3) (see Figure 1).

This clinical trial will be conducted at the Reina Sofía University Hospital of Córdoba (Spain), and the Faculty of Medicine of the University of Córdoba (Spain). This Hospital is reference in the province for cancer care offering to the patients an integral care, including diagnosis, cancer staging, specific oncological treatment, continuous care, palliative care, and disease follow-up. The research group is responsible for the design of the study, the training of the personnel involved in the collection of the data and the application of the interventions, the provision and preparation of all study documents, the supervision of the study, the treatment and analysis of the data obtained, and the communication and dissemination of the results of the investigation.

### Eligibility Criteria

The inclusion criteria for participants are: Men and women, aged between 18 and 70 years/Cancer diagnosis, stages I–III/Cancer type: Breast/Lung/Colorectal/Eligible for or currently receiving cancer treatment/Ability to be fluent in Spanish/Have a smartphone with daily internet access and basic smartphone skills/app handling skills/Not currently participating in another clinical trial/Not currently receiving other psychological treatment.

The exclusion criteria for participants in the trial are: Men and women aged > 70 years/Diagnosis of cancer, stage IV or other types of cancer/Not eligible for treatment/Inability to handle himself fluently in Spanish/Not having a smartphone with daily internet access and/or lack of basic smartphone skills/app handling skills/Currently participating in another clinical trial/Currently receiving other psychological treatment.

### Interventions

The authors have developed a manualized protocol based on the recommendation to implement ACT therapy (Wilson and Luciano, 2014) and previous research with cancer patients (Feros et al., 2013; Arch and Mitchell, 2016). Interventions will be carried out by psychologists with at least master’s degree in clinical psychology, with previous training in psychological intervention in cancer patients and particularly using ACT in these patients. Furthermore, before the intervention starts, the manualized protocol will be supervised by a trained psychologist that works daily with cancer patients. After completing the revision of the manual, the therapist of the trial will receive specific training about how to intervene with cancer patients using ACT. The therapists who are going to carry out the interventions will be supervised by a coordinator with the aim of solving any doubts that may arise during the established coordination meetings.

#### Face-to-Face Acceptation and Commitment Therapy Group

The main objective of the ACT is to develop psychological flexibility, using acceptance strategies, along with mindfulness techniques and different behavioral techniques (Feros et al., 2013). In cancer patients and survivors, ACT helps to cope with situations of distress or fear, and helps people to clarify their values and to commit to living in a more positive way according to them (Arch and Mitchell, 2016). In the face-to-face ACT group, experiential exercises are carried out. Also, metaphors, discussions and assignments are used to promote awareness and flexibility about the thoughts and emotions associated with cancer. This intervention will be carried out in groups of approximately 10 people. It consists of 8 sessions of 1 hour each, which will be developed weekly, over 8 weeks. The sessions will address the key contents, which are organized into 5 specific modules: Psychoeducation and therapeutic relationship; Mindfulness (awareness of distressing thoughts and feelings); Creative hopelessness (contact with the problem and acceptance); The self as context (defusion and deliteralization); Clarification of personal values and commitment to meaningful activities.

Session 1. *Basic principles of the intervention and establishment of the therapeutic relationship.* In this session, the first contact with the group will be established and the basic principles on which the intervention will be based (Mindfulness, relaxation techniques, and components of acceptance and commitment therapy) will be presented. In addition, it will include a brief psychoeducational component on the consequences of the disease in different areas (family, social, work, economy, emotional, and physical).

Session 2. *Psychoeducation and first contact with mindfulness.* This session deepens the previous knowledge of the group on aspects related to cancer: anxiety, depression, insomnia, fatigue, and the experiential avoidance response as coping. The principles on which mindfulness is based will be presented and a brief exercise will be carried out (Raisin exercise).

Session 3. *Creative hopelessness. First contact with the problem and contact with the private events experienced with great discomfort.* The fundamental objective of this session is to establish contact with experiential avoidance. To do so, the principles of acceptance of the present experience assuming that suffering is normal will be described, along with the necessity of questioning the pattern of interaction with the negative aspects of its existence through the creative despair. In addition, a short mindfulness exercise will be carried out: Mindful Walking.

Session 4. *Understanding the importance of a values-based life*. The objective of this fourth session is to begin to clarify the objectives, goals and values that guide the person’s life in a worthwhile direction. It will try to determine what those avoided (even forgotten) values are, the valuable directions to achieve them, and the gradual establishment of a positive and open attitude toward the progressive exposure to the avoided content in relation to what is important for that person.

Session 5. *Defusion and deliteralization of private events “Self-as-context.”* The main objective of this fifth session is to achieve a deactivation of verbal functions or the distancing of the psychological content: to differentiate the domains or dimensions of the self from its behavior (private events or public behavior); differentiate between what is present verbally and what is made present by language; learn to treat thoughts as thoughts, evaluations as evaluations, and memories as memories. It will also include a brief Mindfulness Practice: The Leaves in the River.

Session 6. *Analyze the problem of control and acceptance as an alternative.* The objective of this sixth session is to analyze the usefulness of the cognitive-emotional control strategy; the person experiences the paradox that their actions to solve the problem are giving the opposite result and that the control strategies put in place have threatened their values, thus promoting psychological flexibility with respect to the avoided content.

Session 7. *Values and barriers.* In the seventh session, the focus will be on clarifying the objectives, goals and values that guide the person’s life in a valuable direction. It will try to determine what those avoided (even forgotten) values are, the valuable directions to achieve them, and the gradual establishment of a positive and open attitude toward the progressive exposure to the avoided content in relation to what is important for that person.

Session 8. *Concluding the therapy*. The objective of this last session will be to review the contents and activities carried out to date and resolve any possible doubts that may arise, providing alternatives to cognitive defusion and experiential avoidance.

#### Face-to-Face Acceptation and Commitment Therapy Group + App

The multimodal ACT (ACT + app) follows the same principles described above, and addresses the same key content, but combines face-to-face sessions with non-face-to-face activities and resources, using a mobile application in order to facilitate access, patient adherence and follow-up. The participants randomly assigned to this group will receive the same intervention program as the face-to-face group described before, with the same characteristics, along with interspersed non-face-to-face activities, which will serve as support for each of the sessions. The non-contact activities will consist of metaphors used in ACT programs (i.e., Dirty glass metaphor/Man in the hole metaphor/The unwelcome party guest metaphor), mindfulness and breathing exercises using audio files, and registration of values. These activities will be delivered weekly using a mobile phone application developed by the authors for this research.

#### Waitlist Group

This group will receive the usual care during the essay, but the participants in this group will be offered to receive the face-to-face- ACT group intervention + app once the ACT groups finish the planned intervention and after post-treatment assessment.

Any participant in the intervention groups who meets any of the following criteria will be excluded: Participants’ request to left the study/Participants who stop cancer treatment or change their stage to stage IV/Difficulty to continue with the intervention due to the presence of cancer treatment associated symptoms. Participants who receive other psychological treatment during the trial will be excluded, as stated in exclusion criteria.

During the intervention sessions, the therapist will emphasize the need to attend them on a regular basis and carry out assignments, in addition to highlighting the need to attend the different assessment sessions. For the participants in the ACT face-to-face + app group, the activities to be carried out weekly using the application must be marked by the participants themselves once they are finished, so that the therapist can verify that the activity has indeed been carried out in the established period.

### Outcomes

#### Primary Outcomes

Taking into account the data obtained by previous research in similar studies, it is expected to observe a significant increase in the mean of the scores obtained in psychological flexibility evaluated with the acceptance and action questionnaire-II (AAQ-II) questionnaire in patients who receive the combined intervention face-to-face ACT + app when comparing the scores obtained in T0 (pre-treatment) with T1 (post-treatment). It is also expected that the scores obtained will remain stable in the two follow-up assessment points, T2 (3 months after completion of the intervention) and T3 (6 months after). Also, it is expected that the mean scores in psychological flexibility will be statistically significant higher in the face-to-face ACT + app group than in the face-to face ACT group at the different assessment points. In addition, it is expected to observe significant improvements in psychological flexibility in the group that receives the face-to-face ACT + app intervention compared to the waitlist group at T1, T2, and T3. Finally, it is expected to observe a significant difference in psychological flexibility in the face-to-face ACT group compared to the waitlist group at the aforementioned evaluation moments.

#### Secondary Outcomes

Regarding the results of the secondary measures of the study, it is expected significant increasing scores in quality of life and posttraumatic growth and decreasing scores in anxiety and depression, fatigue and insomnia from T0 to T1 and these scores remain stable in the follow up assessment (T2 and T3) in the face to face ACT + app group. As with psychological flexibility, it is expected that the mean scores of the face-to-face ACT + app group achieve statistically significant higher values in quality of life and posttraumatic growth and significantly lower scores in anxiety, depression, fatigue and insomnia at T1, T2, and T3 when compared with the face-to-face ACT group and the waitlist group. Finally, it is expected that the mean scores of the face-to-face ACT group in quality of life and posttraumatic growth will be significantly higher, and the scores in anxiety, fatigue and insomnia will be significantly lower, when compared with the scores obtained by the waitlist group at the three assessment points.

#### Participant Timeline

**FIGURE 2:**
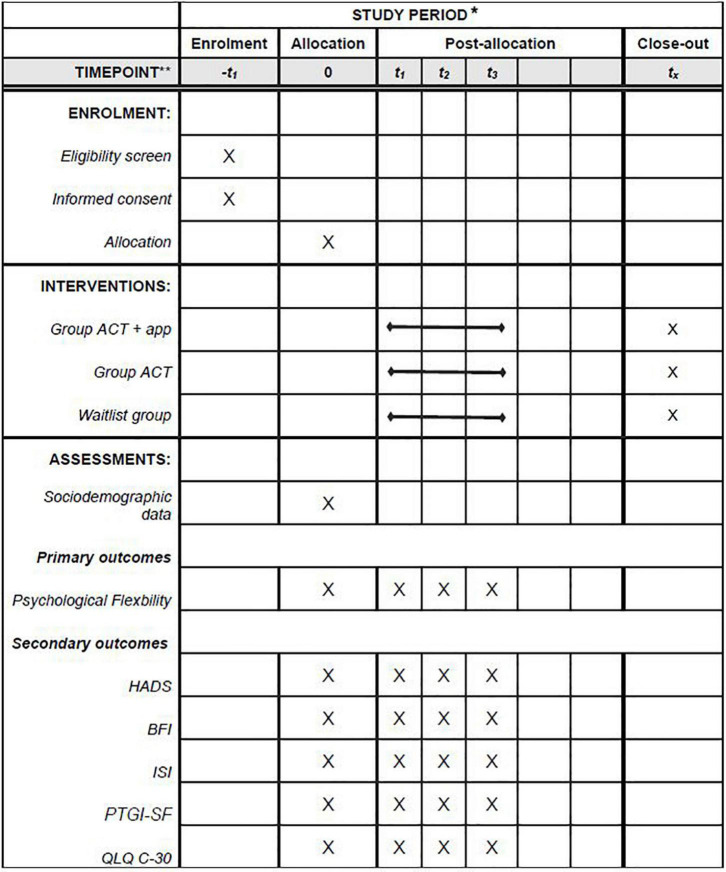
Schedule of enrollment, interventions, and assessments. *Recommended content can be displayed using various schematic formats. See SPIRIT 2013. Explanation and Elaboration for examples from protocols. **List specific timepoints in this row.

Time schedule of recruitment, interventions, assessment, and follow-ups are available in the flow diagram (see Figure 2).

### Sample Size

To determine the optimal sample size, we considered the effect sizes in psychological flexibility obtained in previous works by other authors. A recent meta-analysis (Chunxiao et al., 2020) examined the effect of ACT (including face-to-face and internet-based modalities) on psychological flexibility compared with treatment as usual or waitlist in cancer patients. The results showed a moderate effect size (Hedges’ *g*) of 0.58 at post-treatment. Taking into account these results, a medium effect size of 0.5 (Cohen’s *d*) has been assumed to detect differences between the interventions and waitlist group. Since no software to determine sample size for linear mixed model analyses was available, we used the *f* index of G*Power. Therefore, an effect size of 0.25 (index f) is assumed, equivalent to Cohen’s *d* = 0.5. Thus, with a statistical power of 0.80, an alpha level of 0.05 and a correlation between repeated measures of 0.5, a total sample of 102 participants is required (34 subjects in each group). In order to control the lack of participants during the intervention and evaluation process, based on a previous study (Arch and Mitchell, 2016) we assume a dropout rate of 10%. Consequently, we require to include 112 participants in our study (38 participants per group).

### Recruitment and Assignment to Interventions

Participants will be recruited at the Reina Sofia University Hospital in Córdoba (Spain). The oncology and nursing team will approach to possible participants following the inclusion and exclusion criteria of the protocol during their visits. Eligible participants will sign an informed consent before randomization. Participants will be randomly allocated to one of the three conditions of the study (face-to-face ACT + app/face-to-face ACT/waitlist group) in a ratio of 1:1:1 using computer-generated sets of numbers^1^ and stratified by cancer type (breast/lung/colorectal). Group assignment will be provided to the trial research team in closed opaque envelopes. Randomization and allocation concealment will be carried out by trial independent research staff.

### Blinding

Participants will receive information about the interventions but not about the main objectives of the study and which intervention is considered active. Also, members of the work group of the project involved in the assessment of the participants will be blinded to the objectives of the study and participants assignment.

### Instruments

Sociodemographics characteristics of the participants will be collected using a structured interview, including information about sex, age, marital status, education, professional activity, annual income, cancer type, treatment, and cancer stage. Also, the participants will be asked if they are currently taken psychiatric medication, and, if yes, the type of medication and daily dose. In addition, all the participants will complete the following questionnaires:

#### Acceptance and Action Questionnaire-II

This instrument is designed to assess experiential avoidance and psychological inflexibility through 7 items that are answered with a Likert-type scale from 1 (never true) to 7 (always true), with a higher score indicating greater experiential avoidance. The questionnaire has adequate psychometric properties in its original version (α = 0.84) and in its adaptation to Spanish (α = 0.75–0.93) (Ruiz et al., 2013). It has been widely used in cancer patients (González-Fernández and Fernández-Rodríguez, 2019).

#### Hospital Anxiety and Depression Scale

This scale is used to assess anxiety and depression in hospital settings. It is made up of 14 items (with two subscales; 7 for anxiety and 7 for depression), which are answered on a scale from 0 to 3, with maximum values of 21 in each of the subscales. The cut-off point for establishing the presence of anxiety and depression is set at 8 (probable case). This scale has been adapted and validated into Spanish, showing good psychometric properties (α = 0.86) (Zigmond and Snaith, 1983; Terol-Cantero et al., 2015).

#### Brief Fatigue Inventory

It is an instrument developed to assess fatigue in cancer patients that consists of 10 items with a Likert-type scale that assesses fatigue experienced in the last 24 h from 0 (no fatigue) to 10 (worst fatigue imaginable). It also includes a scale to assess the degree to which fatigue has interfered with the patient’s daily life in the last 24 h, with a scale ranging from 0 (does not interfere) to 10 (completely interfered). The recommended cut-off points in cancer patients are: 1–3 (mild); 4–7 (moderate) and 8–10 (severe) (Chang et al., 2007). The scale has adequate psychometric properties in its original version (α = 0.96) and in its adaptation to Spanish (α = 0.97) (Mendoza et al., 1999; Lorca et al., 2016).

#### Insomnia Severity Index

This instrument is designed to assess the severity of sleep onset, sleep maintenance and early morning awakening problems along with satisfaction with sleep pattern, interference with daily functioning, noticeability of impairment attributed to the sleep problem by others, and degree of distress or concern related with the sleep problem through 7 items that are scored on a scale from 0 (not at all) to 4 (very severe/very dissatisfied/very much). The sum of the scores given by the subject to the different items results in a total score that ranges from 0 to 28. The recommended cut-off points are as follows: 0–7 (absence of clinical insomnia), 8–14 (subclinical insomnia), 15–21 (moderate clinical insomnia), and 22–28 (severe clinical insomnia). The psychometric properties of the instrument are adequate in its original version (α = 0.91) and in its adaptation to Spanish (α = 0.82) (Bastien et al., 2001; Fernández-Mendoza et al., 2012).

#### Post-traumatic Growth Inventory-Short Version

It is an instrument designed to evaluate the positive change that people who are subjected to a traumatic event may experience, using 10 items that respond to a Likert-type scale from 0 (I have not experienced that change) to 5 (I have experienced that change to a great extent), with higher scores showing greater positive change. The instrument has good psychometric properties in its original version (α = 0.90) and in its adaptation to Spanish (α = 0.83) (Tedeschi and Calhoun, 1996; Castro et al., 2015).

*EORTC QLQ C-30 (version 3).* This is an instrument developed to assess the quality of life in cancer patients using 30 questions that refer to the quality of life experienced by the patient during the last week. The first 28 items include questions about different symptoms and are answered on a scale that ranges from 1 (not at all) to 4 (a lot), while the last two ask patients about the perception of their global health and quality of life in a scale from 1 (terrible) to 7 (excellent). The different items are grouped into the functional subscale (physical/role/emotional/cognitive/social and global) and the symptom subscale (fatigue/nausea and vomiting/dyspnea/sleep problems/loss of appetite/constipation/diarrhea and financial impact). This instrument has been developed to be applied in cancer patients obtaining good psychometric properties in its version in Spanish (α > 0.70) (Aaronson et al., 1993).

In addition to the scales and questionnaires described above, a specific scale will be included to consult the participants about their satisfaction with the intervention received in the present study. In order to ensure that participants will complete the follow up assessments, the psychotherapists will contact by phone to the participants monthly after the intervention ends to remind them the necessity to complete the planned assessments.

### Data Management

To ensure the anonymity, a code number will be assigned to each participant. Participants’ responses to the sociodemographic data and questionnaires will be collected on paper and kept in a locked filing cabinet during and after the trial ends. The mobile application that will be used in this project does not collect personal information from the end user, nor from the terminal on which it is installed, nor from the history or content of its internal memory. The purpose of the application is an additional aid to therapeutic treatment, offering a series of activities and exercises, from which only information is collected on whether they have been performed or not. Access to the application is not available for all users, although it will be hosted in the main stores in the market. The user/patient enters by identifying themselves with a tracking code that only their therapist knows, as otherwise they could not be tracked effectively. The information that can be stored (never of a personal nature) will be synchronized with a MySQL database hosted on the servers of the University of Córdoba, which have high security and stability standards. Access directly from mobile terminals to the database is not allowed, precisely in compliance with rigorous security protocols, so it is done through web services executed on the machine itself, within the secure environment of the University of Córdoba.

### Statistical Methods

Data will be analyzed following the intention to treat (ITT) and per protocol approaches. First, ANOVA or chi-square will be performed to compare the sociodemographic variables and the outcome measures at baseline (T0). Second, to examine the longitudinal changes over time (baseline, post-treatment and follow-ups) and the differences between groups in the variables under study, we will use mixed linear models, since these models are more precise than the repeated measures ANOVA (Gueorguieva and Krystal, 2004). In ITT analysis (all randomized patients are included in the analysis), incomplete or missing data will be considered using the maximum likelihood estimation method. In addition, the effect sizes will be calculated by Cohen’s *d* (bias corrected) (Hedges, 1981) as a measure of the differences between the Standardized Mean Changes (SMC) (T0-T1; T0-T2; T0-T3) of the respective groups (Becker, 1988). We will use the formula *d* = *c* ⋅ ([*M*_*pre*_ –*M*_*post*_]/*SD*_*pre*_) to obtain the SMC, where *c* is the bias correction factor, *M*_*pre*_ and *M*_*post*_ are the means of the pre–test and post–test scores, respectively, and *SD*_*pre*_ is the pre–test standard deviation score (Morris and DeShon, 2002). The SMC will be calculated for each group, providing the *d* index of the general effect size from the differences between them (Face-to-face ACT group vs. Face-to-face ACT group + App; Face-to-face ACT group vs. Waitlist group; Face-to-face ACT group + App vs. Waitlist group). Once the analyses have been performed, summary tables will be displayed for all the planned evaluations: pre-treatment (T0), post-treatment (T1), follow-up at 3 months (T2) and follow-up at 6 months (T3). The results will show frequencies and descriptive statistics (the mean, standard deviation and percentages) to summarize the characteristics of both the total sample and the participants that make up each of the three groups. Finally, the results of the study will be shown in accordance with the 2010 statement of the Consolidated Standards of Reporting Trials (CONSORT) (Moher et al., 2010; Schulz et al., 2010).

### Monitoring

Due to the nature of this trial, a data monitoring committee (DMC) is not necessary as the planned intervention is short (8 weeks) and there are no associated risks to participate in the different interventions planned. Also, we do not expect any harms or adverse effects during the intervention.

This trial will be monitored by an independent committee from the Andalusian Biomedical Research Ethics. This committee asks to the researcher to complete a document with information about the trial, including the number of participants recruited, the participants who withdraw their participation of the study and others. This document will be completed and send to the committee for evaluation once a year. Relevant changes to the approved protocol will be notified to the review board of the Andalusian Biomedical Research Ethics during the annual revision of the protocol.

### Ethics Approval and Consent to Participate

This research obtained the approval from the Andalusian Biomedical Research Ethics (ref. number: 9050) and it is registered with the international standard randomized controlled trial number: NCT05126823.^2^ All the participants of the study will be provided with both oral and written information about the study and will sign an informed consent that states the confidentiality of the data prior the study begin.

Once the oncologists and the nursing team have selected the potential study participants following the inclusion and exclusion criteria described previously, the participants will be directed to a nurse professional and a trained psychologist with experience in cancer patients who will inform them about the characteristics of the study and will respond to questions that participants may have about their participation (e.g., type of intervention, duration, cost, safety, etc.), using an information sheet with trial details. If the participant agrees to continue, they will be provided with an informed consent which states their consent to participate in the trial, the possibility of leaving the study at any time without affecting their medical care and without having to provide any explanation and guarantee the confidentiality of the results obtained. The informed consent will be stored in a close cabinet in the Hospital. The participant information will be coded with an ID number to maintain the confidentiality, and all personal information of the participants will be stored in a locked file with limited access only available to the nurse team in the Reina Sofía University Hospital. The research team of the protocol will be given access to the cleaned data sets. As it is stated before, in this dataset the participant information will be coded with an ID number. Project data sets will be stored in a locked computer acquired for this purpose and the data will be password protected to ensure that only approved members of the research team will have access to this data.

## Discussion

In cancer patients, it is common for psychological symptoms associated with the course of the disease to appear, such as anxiety, depression, insomnia, fatigue, and a poorer quality of life (Mock et al., 2000; Satin et al., 2009; Corbett et al., 2016; Kuhnt et al., 2016; Zhou et al., 2017; Pitman et al., 2018; De Mol et al., 2020; Erim et al., 2020; Park et al., 2020; Taarnhøj et al., 2020; Yun et al., 2021). Among the different interventions developed to reduce these symptoms in cancer patients, acceptance and commitment therapy performs better than other interventions (such as cognitive-behavioral therapy) as it focuses on improving the psychological flexibility of patients, which causes improvements in associated symptoms, such as those mentioned above (Feros et al., 2013; Arch and Mitchell, 2016; Aguirre-Camacho and Moreno-Jiménez, 2017; Bas and Dirik, 2019; González-Fernández and Fernández-Rodríguez, 2019). In this sense, the ACT-based interventions carried out including face-to-face and non-face-to-face activities using different formats have shown promising results in previously conducted studies. However, these positive results have to be confirmed with randomized trials with an adequate level of control (Mosher et al., 2018; Baumeister et al., 2014; Hawkes et al., 2014).

This protocol provides information on a randomized and controlled trial in which it is planned to carry out an intervention based on ACT in person and in a group and also uses an app to carry out reinforcement activities between sessions. It is expected that the participants assigned to this group obtain statistically significant better results in psychological flexibility and in the rest of the symptoms included in this study (anxiety, depression, quality of life, fatigue, insomnia, and post-traumatic growth) than the remaining groups once the intervention is completed. In addition, significant improvements are expected to be observed in the face-to-face ACT group compared to the waitlist control group.

One of the strengths of this protocol is the inclusion of a significant number of participants, which results from the estimation of the adequate sample size to obtain significant results based on previous studies. Also, the inclusion of cancer patients with different diagnoses can help to broaden the knowledge about the efficacy of ACT in oncological settings, which may increase its applicability. Furthermore, the use of a randomized, controlled, two-follow-up design can help establish the long-term efficacy of the intervention.

Notwithstanding, it is necessary to mention some of the limitations that may appear when carrying out this study. First, the inclusion of patients with different diagnoses and treatments may influence the results obtained, since there may be different strategies and effects of the treatments that influence the results. Furthermore, to avoid loss of participants in the waitlist group, the intervention in this group will start immediately after completion in the two ACT groups and this circumstance may limit the information about the long-term effects of the intervention.

Despite these limitations, the results that are expected to be obtained after conducting this trial may be useful in helping patients during their treatment to reduce symptoms that appear frequently during the course of the disease and that can negatively affect their daily life.

## Ethics Statement

The studies involving human participants were reviewed and approved by Andalusian Biomedical Research Ethics (ref. number: 9050). The patients/participants provided their written informed consent to participate in this study.

## Author Contributions

FG-T conceived of the study and was the grant holder. FG-T, RC-M, BL-S, MJ-M, SR, EM, JM, MJ, FC-H, MG-L, and EA initiated the study. MG-L and JM provided statistical expertise in clinical trial design. All authors contributed to refinement of the study protocol and approved the final manuscript.

## Conflict of Interest

The authors declare that the research was conducted in the absence of any commercial or financial relationships that could be construed as a potential conflict of interest.

## Publisher’s Note

All claims expressed in this article are solely those of the authors and do not necessarily represent those of their affiliated organizations, or those of the publisher, the editors and the reviewers. Any product that may be evaluated in this article, or claim that may be made by its manufacturer, is not guaranteed or endorsed by the publisher.

## Footnotes

1
https://www.randomizer.org/

2
www.clinicaltrials.gov
